# Non-contrast microvascular flow imaging in abdominal ultrasound: a pictorial essay

**DOI:** 10.1007/s40477-026-01163-7

**Published:** 2026-05-14

**Authors:** Marco Musmeci, Alice Brighenti, Carla Serra, Andrea Boccatonda

**Affiliations:** Diagnostic and Therapeutic Interventional Ultrasound Unit, IRCCS Azienda Ospedaliero-Universitaria Di Bologna, Policlinico Sant’Orsola-Malpighi, Via Massarenti N 9, 40138 Bologna, Italy

**Keywords:** Microvascular flow imaging, MV-Flow, Abdominal ultrasound, Vascular pathology, Portal hypertension, Pyelonephritis

## Abstract

The evaluation of low-velocity blood flow and complex vascular patterns remains a recognized limitation of conventional color Doppler ultrasound in abdominal imaging. Microvascular flow imaging (MV-Flow) is an advanced Doppler technique designed to enhance sensitivity to slow and heterogeneous blood flow while effectively suppressing tissue motion artifacts. This pictorial essay illustrates the main sonographic applications of MV-Flow across a wide spectrum of abdominal clinical scenarios, including arterial flow alterations, venous and portal vascular disorders, post-transplantation hemodynamic changes, inflammatory parenchymal conditions, and selected focal liver lesions. Representative imaging examples are provided to highlight characteristic flow patterns and vascular architectures that are not readily appreciable with conventional Doppler techniques. In this context, MV-Flow represents a valuable complementary tool in abdominal ultrasound, improving pattern recognition and diagnostic confidence during non-invasive clinical assessment. In addition, by improving the non-invasive characterization of vascular and perfusion patterns, MV-Flow may reduce the need for further cross-sectional imaging, potentially lowering healthcare costs and minimizing patient exposure to ionizing radiation.

## Introduction

Ultrasound plays a central role in abdominal imaging thanks to its wide availability, real-time capability, and absence of ionizing radiation. Recent ESC and ACC/AHA guidelines on vascular diseases emphasize the increasing role of non-invasive, cost-effective imaging modalities, including ultrasound, as first-line tools for the assessment and follow-up of vascular pathologies [1, 2]. In this evolving scenario, advanced Doppler techniques such as MV-Flow may further enhance the diagnostic capabilities of conventional ultrasound. Conventional Color and Power Doppler techniques are routinely used to assess vascularization and blood flow; however, their diagnostic performance may be limited in clinical scenarios characterized by low-velocity flow, complex hemodynamics, or subtle microvascular alterations [3]. Angle dependency, blooming artifacts, and reduced sensitivity to slow flow may hinder confident interpretation in selected abdominal conditions. Recent advances in ultrasound technology have led to the development of microvascular flow (MV-flow) imaging techniques designed to improve the visualization of low-velocity blood flow and microvascular structures [4–8]. MV-Flow is a Doppler-based technique that enhances sensitivity to slow-flow signals while reducing motion-related artifacts, allowing more detailed depiction of vascular and perfusion patterns without the use of contrast agents [4, 9]. In abdominal ultrasound, the ability to visualize microvascular flow patterns has relevant clinical implications across a wide range of conditions, including arterial flow alterations, venous and portal abnormalities, inflammatory perfusion disorders, and focal liver lesions [6, 10]. In these settings, MV-Flow may provide complementary information to conventional Doppler techniques, supporting pattern recognition and diagnostic confidence during routine examinations [10]. This pictorial essay aims to illustrate the potential clinical applications of MV-Flow in abdominal ultrasound through representative imaging examples, highlighting characteristic vascular and perfusion patterns that are not readily appreciable with standard Doppler techniques.

## Technical overview of MV-Flow

MV-Flow is an advanced Doppler-based ultrasound technique developed to improve the visualization of low-velocity blood flow and microvascular structures [4, 5, 10]. The technique is based on dedicated signal processing strategies that enhance sensitivity to slow-flow components while effectively suppressing tissue motion and clutter artifacts, which commonly limit conventional color and power Doppler imaging [4, 6]. A key feature of MV-Flow is its ability to preserve low-amplitude flow signals arising from small vessels and microcirculation, allowing depiction of flow patterns that are often masked by surrounding tissue motion or noise on standard Doppler modalities [4, 5, 9]. Unlike conventional Doppler techniques, which primarily emphasize velocity information, MV-Flow is optimized for qualitative assessment of flow distribution and vascular architecture, facilitating recognition of characteristic flow patterns [4, 6]. In abdominal ultrasound, these technical characteristics translate into improved depiction of complex and heterogeneous flow conditions, such as peri-prosthetic slow flow, venous recanalization, and parenchymal perfusion abnormalities [10]. By providing real-time, high-sensitivity visualization of microvascular flow without the need for contrast agents, MV-Flow complements conventional Doppler techniques and supports pattern-based interpretation during routine abdominal examinations.

Although microvascular flow imaging techniques share the common goal of improving visualization of low-velocity blood flow, important differences exist among commercially available implementations, which are largely based on proprietary signal processing algorithms [10]. This “black box” nature may limit direct comparison and standardization across ultrasound systems, and standardized terminology is currently lacking across vendors. Canon’s Superb Microvascular Imaging (SMI) is based on advanced spatiotemporal filtering techniques that separate slow-flow signals from tissue motion, allowing high-sensitivity visualization of microvascular flow while maintaining high spatial resolution [10, 11]. GE Healthcare has developed multiple approaches to improve visualization of slow and complex blood flow. B-Flow is a non-Doppler technique based on coded excitation and tissue–blood equalization, enabling direct visualization of blood reflectors [3, 12]; however, it is not specifically optimized for microvascular flow detection. Recent implementations include Doppler-based microvascular imaging solutions (MVI) designed to enhance sensitivity to low-velocity flow. Samsung’s MV-Flow employs adaptive filtering algorithms and high-frame-rate processing to enhance detection of low-velocity and multidirectional flow, enabling detailed visualization of small vessels and microvascular architecture [13, 14]. Philips has introduced microvascular flow imaging (MFI) techniques based on proprietary clutter suppression algorithms to improve detection of low-speed blood flow, particularly in tumor vascularity assessment [15, 16]. Supersonic Imagine (Angio PLUS) leverages ultrafast plane-wave imaging to achieve very high frame rates, enabling accumulation of flow signals and improved sensitivity to microvascular flow [17]. Other vendors, including Esaote (microV) and Mindray (UMA), have also developed microvascular imaging solutions based on advanced Doppler signal processing aimed at improving detection of slow and small-vessel flow [18, 19]. Despite these technological differences, variability in implementation, image appearance, and sensitivity remains a limitation, and caution is required when extrapolating findings across different platforms. These differences may affect image interpretation and limit reproducibility across platforms.

## Clinical applications

Microvascular flow imaging is particularly well suited for evaluating abdominal vascular conditions characterized by low-velocity, complex, or dynamically changing blood flow. In these scenarios, conventional color and power Doppler techniques may fail to depict subtle vascular signals because of limited sensitivity, angle dependency, or motion-related artifacts. MV-Flow enhances the visualization of slow-flow vessels and microvascular structures, providing additional hemodynamic information across different abdominal clinical settings.

### Focal liver lesion

**Fig. 1:**
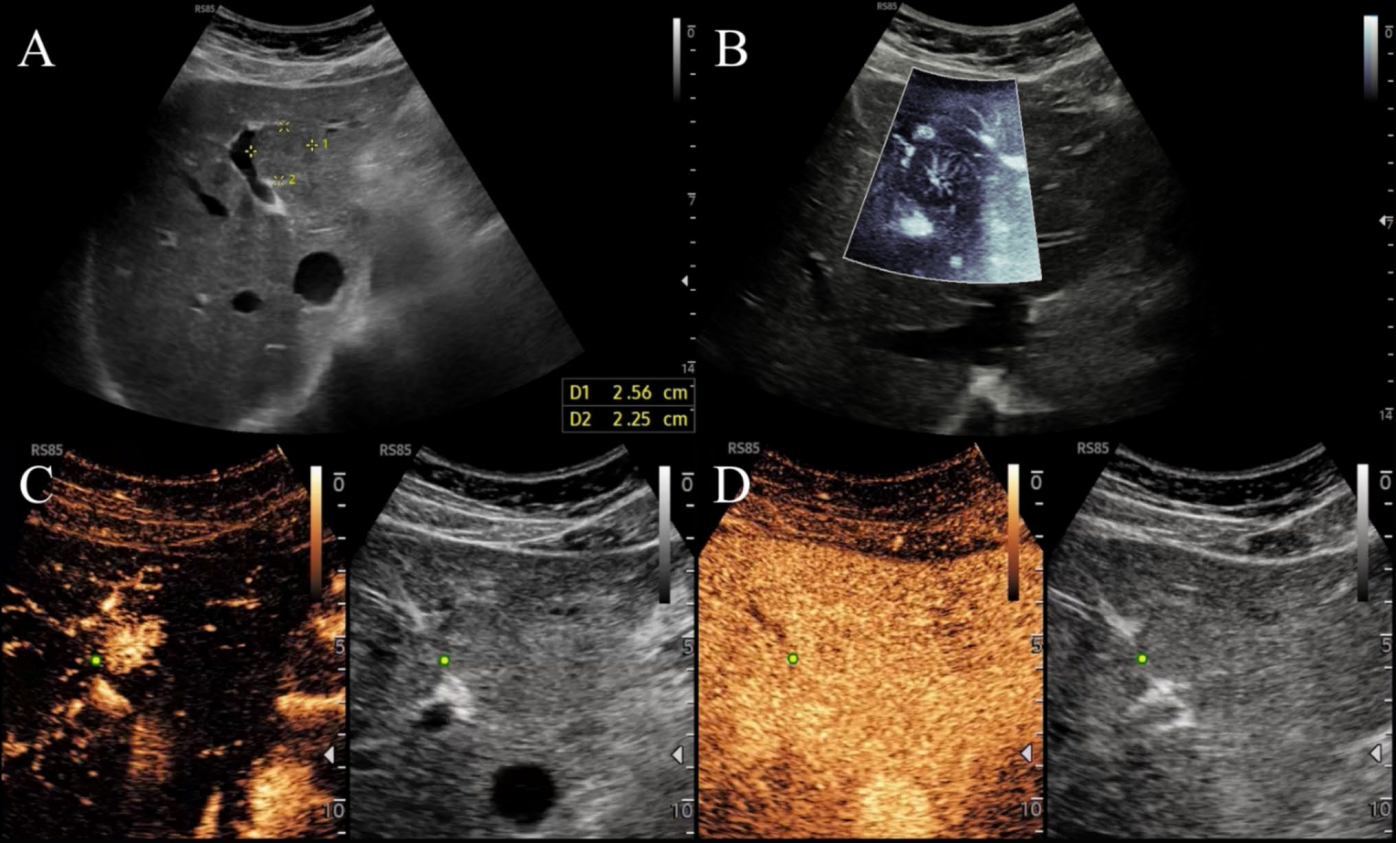
Focal nodular hyperplasia: multimodal ultrasound assessment **A** B-mode ultrasound demonstrates a well-defined focal hepatic lesion without specific morphological features, allowing confident characterization. **B** MV-Flow imaging depicts a characteristic radial intralesional vascular pattern, consistent with the typical spoke-wheel architecture of focal nodular hyperplasia. **C** Contrast-enhanced ultrasound (CEUS) arterial phase shows intense and homogeneous hyperenhancement of the lesion with a centrifugal filling pattern. **D** CEUS late phase demonstrates sustained isoenhancement relative to the surrounding liver parenchyma, confirming the benign vascular behavior of focal nodular hyperplasia

Focal nodular hyperplasia (FNH) is a benign hepatic lesion characterized by a distinctive vascular architecture, typically composed of a central feeding artery with radially arranged vessels. The recognition of this vascular pattern plays a key role in the non-invasive characterization of FNH at ultrasound. Conventional color and power Doppler ultrasound may demonstrate arterial signals within the lesion; however, depiction of the complete vascular architecture can be limited by angle dependency, blooming artifacts, or insufficient sensitivity to low-velocity intralesional flow. These limitations may hinder confident pattern recognition, particularly in small or deeply located lesions. MV-Flow enhances visualization of intralesional microvascular architecture by clearly depicting the radial and spoke-wheel–like vascular arrangement typical of FNH (Fig. 1). In contrast, malignant lesions such as hepatocellular carcinoma typically demonstrate a more irregular and disorganized intra-tumoral vascular pattern, often described as a “basket” or “vessels-in-tumor” appearance, reflecting chaotic neo-angiogenesis [15, 16]. The improved sensitivity to low-velocity flow supports a more comprehensive assessment of lesion vascularity, facilitating pattern recognition and increasing diagnostic confidence during baseline abdominal ultrasound evaluation. In this context, MV-Flow serves as a valuable complementary tool in the characterization of focal liver lesions.

### Pancreatic solid lesions

**Fig. 2:**
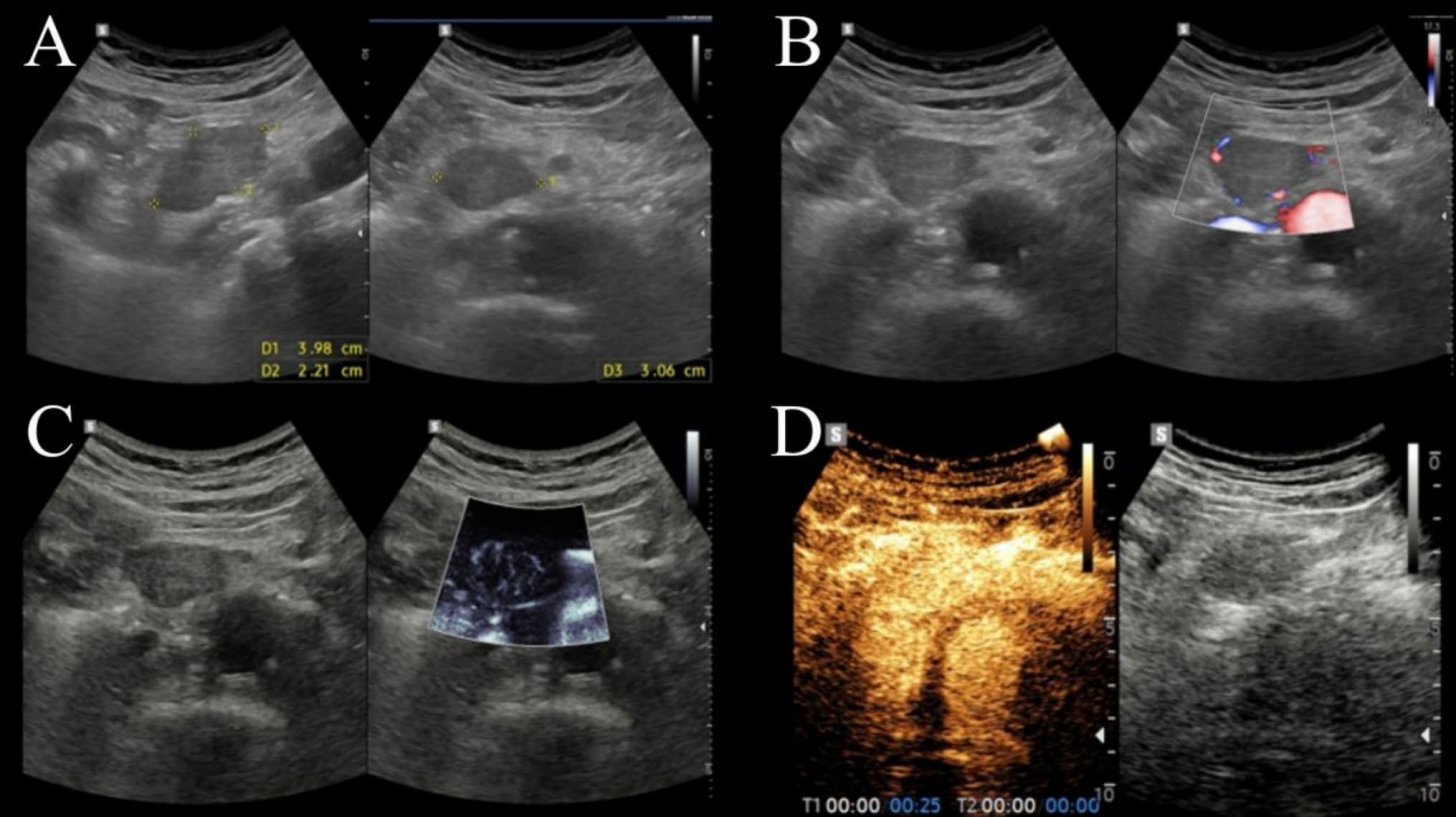
Pancreatic Neuroendocrine tumor: multimodal ultrasound assessment:** A** B-mode ultrasound demonstrates a solid pancreatic mass with heterogeneous echotexture **B** Conventional color Doppler ultrasound fails to demonstrate significant intralesional vascular signals, suggesting absent or minimal detectable blood flow with standard Doppler sensitivity. **C** MV-Flow imaging, in contrast, clearly depicts intralesional microvascular flow, revealing a rich internal vascular pattern not appreciable with conventional color Doppler. The ability to visualize this prominent intralesional vascularization supports differentiation of hypervascular pancreatic neuroendocrine tumor from hypovascular pancreatic adenocarcinoma. **D** Contrast-enhanced ultrasound confirms the presence of intralesional vascularization, supporting the MV-Flow findings and allowing confident differentiation from hypovascular pancreatic lesions

Solid pancreatic lesions represent a diagnostic challenge at baseline abdominal ultrasound, as B-mode imaging often lacks specific morphological features to allow confident lesion characterization. In this setting, evaluation of lesion vascularity plays a key role in the differential diagnosis between hypervascular and hypovascular pancreatic tumors. However, conventional color and power Doppler ultrasound frequently fail to demonstrate intralesional blood flow because of limited sensitivity to slow and microvascular signals, particularly in small or deeply located lesions. MV-Flow enhances the visualization of intralesional microvascular architecture by detecting low-velocity blood flow that is not appreciable with standard Doppler modalities (Fig. 2). This improved sensitivity allows depiction of rich intralesional vascular patterns, supporting pattern recognition during baseline ultrasound evaluation. In particular, visualization of prominent intralesional vascularization may aid in differentiating hypervascular pancreatic neuroendocrine tumors from hypovascular pancreatic adenocarcinoma, which typically shows reduced or absent internal vascular signals at ultrasound. In this context, MV-Flow acts as a valuable complementary technique to conventional Doppler imaging, guiding further diagnostic work-up with contrast-enhanced ultrasound or cross-sectional imaging and improving diagnostic confidence during routine abdominal ultrasound examinations.

### Venous and portal flow abnormalities

Venous and portal vascular disorders represent a common source of diagnostic uncertainty in abdominal ultrasound, particularly in conditions characterized by slow, fragmented, heterogeneous, or abnormally redistributed blood flow. These hemodynamic patterns may occur during partial or resolving thrombotic events, in the presence of collateral venous networks, after liver transplantation, or in association with abnormal arteriovenous communications. In such settings, conventional color and power Doppler techniques may provide limited information because of reduced sensitivity to low-velocity flow and difficulties in depicting complex venous hemodynamics. MV-Flow enhances the visualization of slow venous flow and abnormal venous pathways, supporting a more detailed assessment of portal, mesenteric, and systemic venous circulation abnormalities.

#### Venous thrombosis and recanalization

**Fig. 3:**
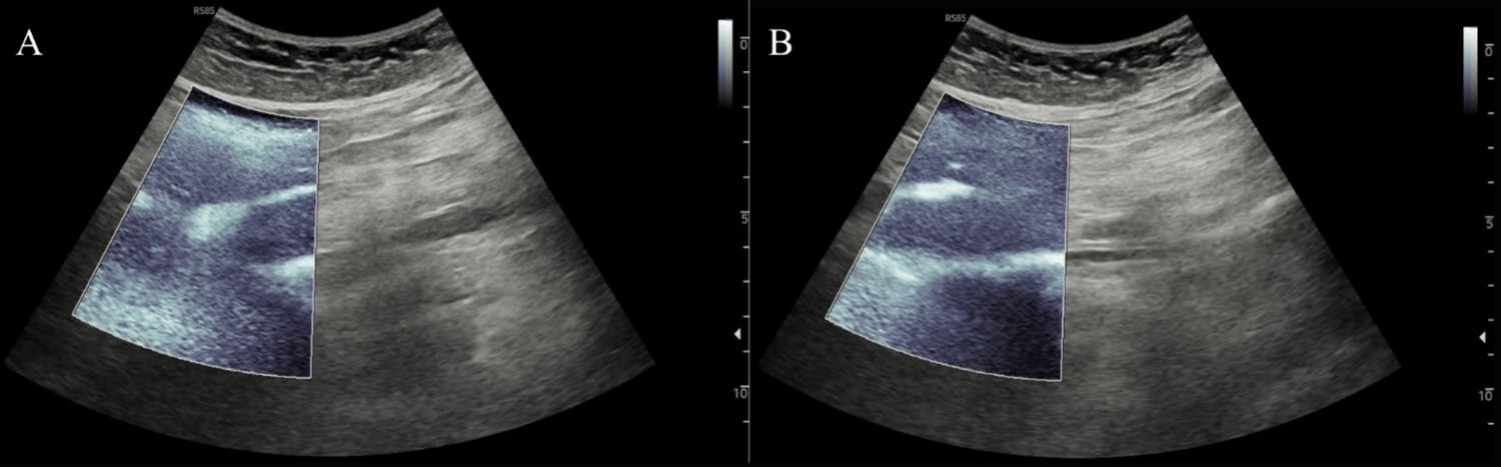
Recanalization of mesenteric venous thrombosis – MV-Flow imaging.** A**, **B** MV-Flow imaging demonstrates continuous low-velocity blood flow within a recanalized mesenteric vein. The technique allows clear visualization of intraluminal venous flow along the vessel course, consistent with venous patency after thrombotic occlusion. This low-velocity flow pattern, difficult to appreciate with conventional Doppler techniques, highlights the ability of MV-Flow to depict venous recanalization during non-invasive follow-up of mesenteric venous thrombosis

**Fig. 4:**
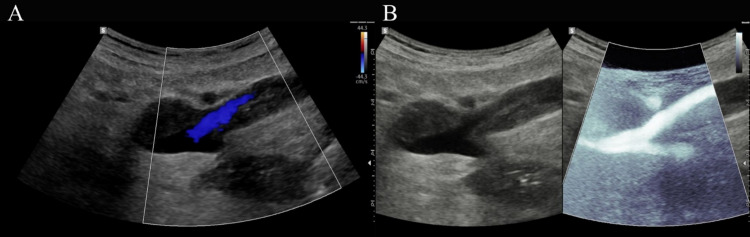
Recanalization of mesenteric venous thrombosis: comparison between conventional Doppler and MV-Flow. **A** Conventional color Doppler ultrasound demonstrates limited and incomplete visualization of venous blood flow within a partially recanalized mesenteric vein, with difficulty in delineating the residual lumen. **B** MV-Flow imaging provides a clearer depiction of low-velocity intraluminal blood flow, allowing direct visualization of the residual venous lumen and the recanalization channels along the vessel course. The enhanced sensitivity of MV-Flow to slow venous flow improves characterization of partial venous recanalization during non-invasive ultrasound follow-up

**Fig. 5:**
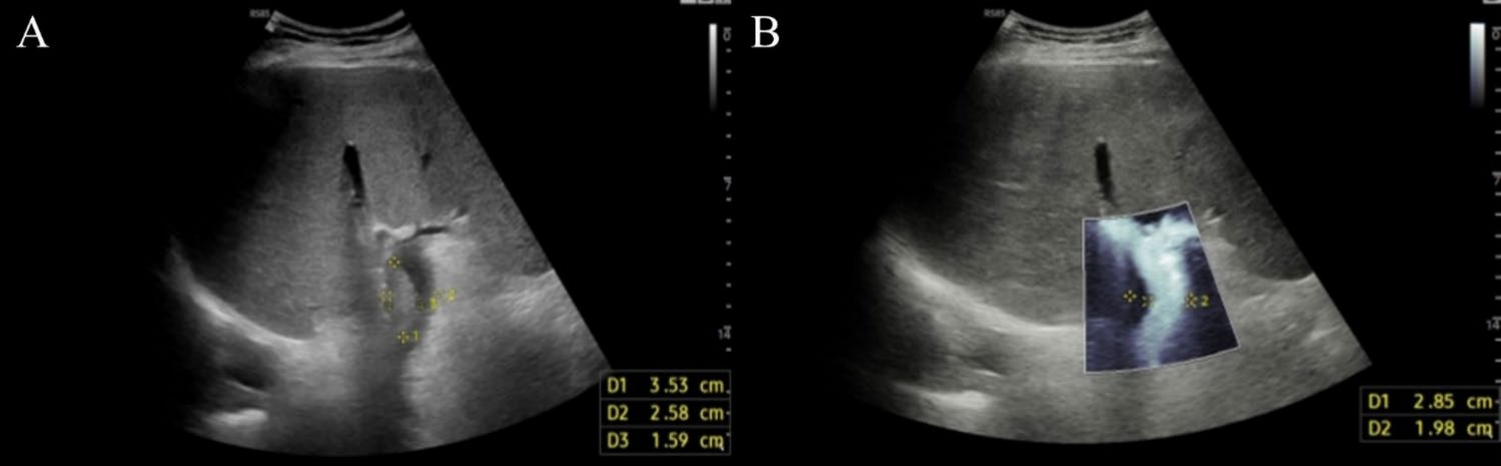
Partial portal vein thrombosis: comparison between B-mode and MV-Flow imaging. **A** B-mode ultrasound demonstrates partial thrombosis of the main portal vein extending into the right portal branch, appearing as intraluminal echogenic material with incomplete lumen occupation. **B** MV-Flow imaging clearly depicts low-velocity residual intraluminal blood flow adjacent to the thrombotic material, allowing direct visualization of the residual patent lumen. The enhanced sensitivity of MV-Flow to slow venous flow facilitates differentiation between partial and complete portal vein thrombosis, improving confidence during non-invasive ultrasound assessment and follow-up

**Fig. 6:**
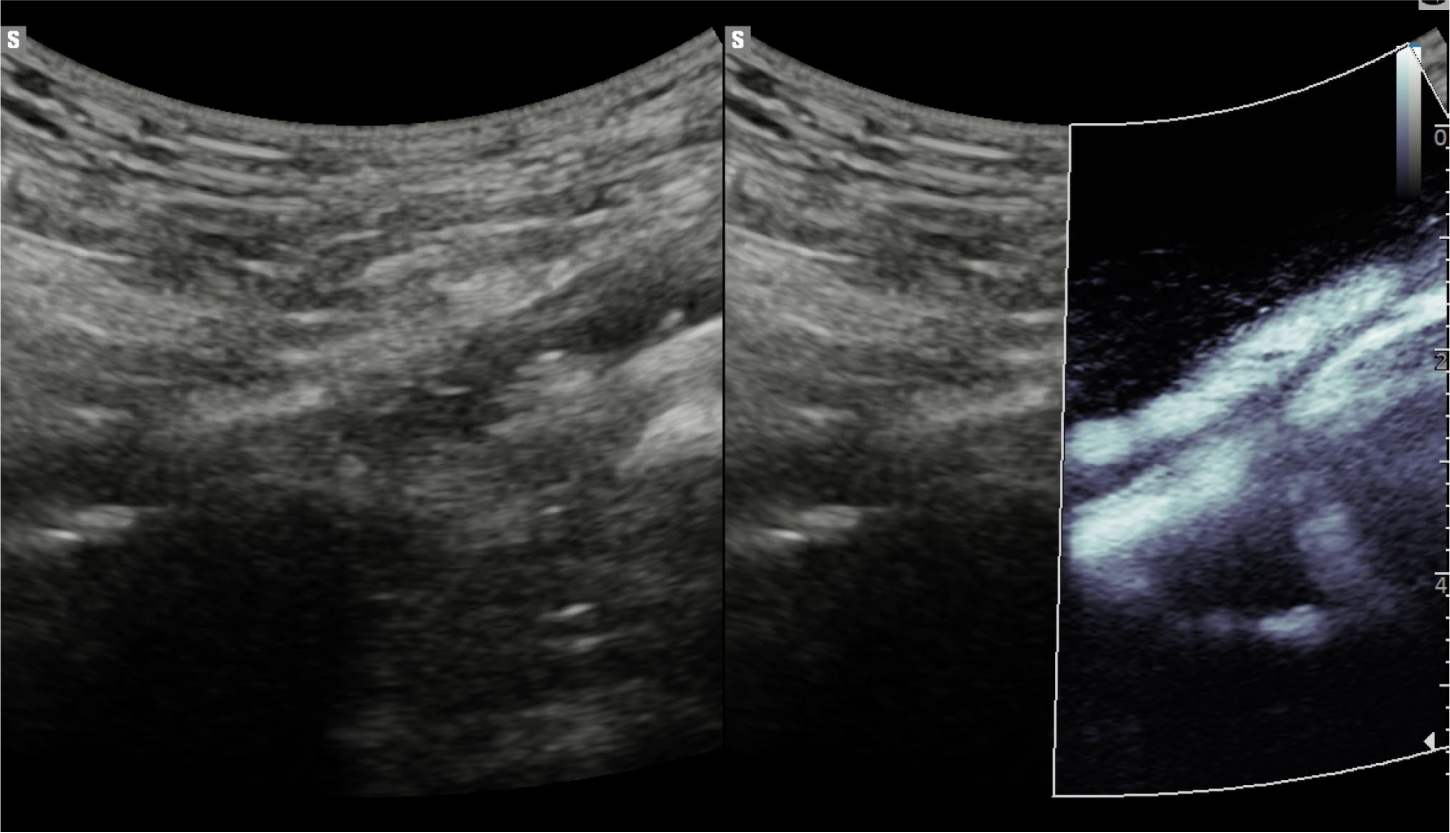
Partial thrombosis of the external iliac vein: comparison between B-mode and MV-Flow imaging. **A** B-mode ultrasound demonstrates intraluminal thrombotic material within the external iliac vein, appearing as an echogenic structure partially occupying the vessel lumen. **B** MV-Flow imaging delineates low-velocity residual venous blood flow adjacent to the thrombotic material, allowing direct visualization of the residual patent lumen. The thrombotic plaque appears as a non-vascularized “dark” area, clearly distinguished from the surrounding venous flow. This improved depiction facilitates differentiation between partial and complete venous thrombosis during non-invasive ultrasound assessment

Venous thrombosis involving the portal, mesenteric, or systemic venous circulation is frequently characterized by slow, fragmented, or eccentric blood flow, particularly during partial thrombosis or recanalization phases [20]. In these conditions, residual intraluminal flow may be confined to narrow channels along the vessel wall, resulting in heterogeneous and low-velocity hemodynamics that are often difficult to detect with conventional color and power Doppler ultrasound. MV-Flow improves the visualization of residual venous blood flow by enhancing sensitivity to low-velocity signals, allowing clearer delineation of the residual lumen and recanalization channels within partially thrombosed veins (Fig. 3). This capability facilitates differentiation between complete and partial venous occlusion and supports identification of subtle intraluminal flow patterns that may not be readily appreciable with standard Doppler techniques (Fig. 4). By enabling depiction of low-velocity venous flow during thrombotic and recanalization phases, MV-Flow contributes to a more confident non-invasive assessment of venous patency during ultrasound follow-up, both in abdominal vessels such as the portal and mesenteric veins and in extra-abdominal districts, including the iliac venous system (Figs. 5 and 6).

#### Portal vein dilation in transplanted patients

**Fig. 7:**
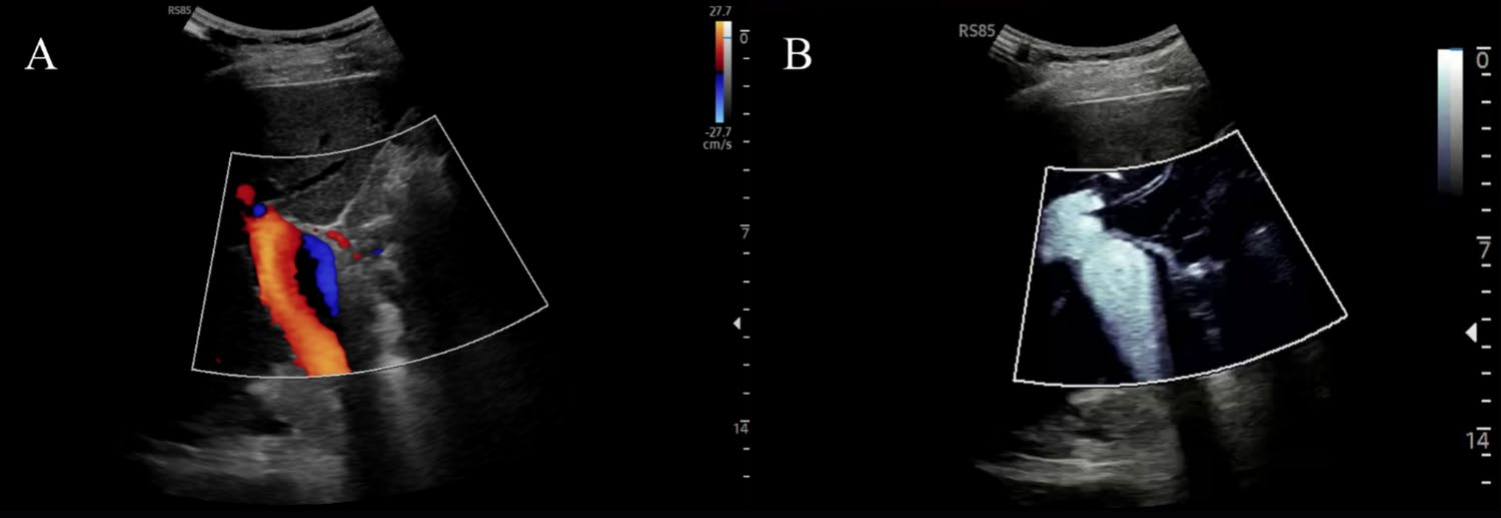
Portal vein dilation in a liver transplant recipient.** A** Conventional color Doppler ultrasound demonstrates portal vein dilation; however, flow depiction appears incomplete and heterogeneous, limiting accurate assessment of portal venous hemodynamics. **B** MV-Flow imaging provides a clearer and more homogeneous visualization of low-velocity portal venous flow, allowing improved depiction of intraluminal flow distribution within the dilated portal vein

Portal vein dilation represents a frequent finding in liver transplant recipients and may be associated with complex hemodynamic alterations, including reduced flow velocity, turbulence, or segmental flow abnormalities. In this setting, conventional color and power Doppler ultrasound may be limited in accurately depicting slow or heterogeneous portal venous flow, particularly in the presence of postoperative anatomical changes. MV-Flow enhances the visualization of low-velocity portal venous flow by depicting subtle intraluminal and periportal flow patterns that may not be readily appreciable with standard Doppler techniques (Fig. 7). The improved sensitivity to slow-flow signals allows a more detailed assessment of portal hemodynamics, supporting the identification of residual or redistributed portal flow in transplanted livers. The ability to directly visualize complex portal flow patterns may contribute to a more confident sonographic evaluation of portal vein dilation in post-transplant patients during routine, non-invasive ultrasound follow-up.

#### Portal cavernoma and collateral venous pathways

**Fig. 8:**
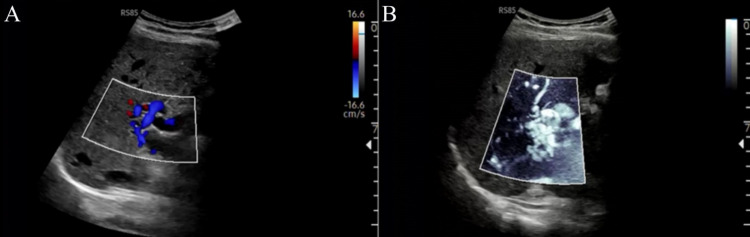
Portal cavernoma: comparison between conventional color Doppler and MV-Flow imaging. **A** Conventional color Doppler ultrasound demonstrates fragmented and limited flow signals within periportal collateral vessels, providing an incomplete depiction of the cavernomatous venous network. **B** MV-Flow imaging clearly visualizes a complex network of tortuous periportal collateral veins with low-velocity blood flow, allowing comprehensive depiction of the cavernomatous transformation. The enhanced sensitivity of MV-Flow to slow and heterogeneous venous flow facilitates recognition of the characteristic cavernoma pattern and improves diagnostic confidence during non-invasive ultrasound evaluation

Portal cavernoma represents the chronic sequela of long-standing portal vein thrombosis and is characterized by the development of a complex network of tortuous collateral veins replacing the normal portal vein lumen. Blood flow within these collateral pathways is typically slow, multidirectional, and heterogeneous, making its depiction challenging with conventional color and power Doppler ultrasound techniques. In this setting, MV-Flow enhances visualization of low-velocity flow within the cavernomatous venous network by improving sensitivity to slow and fragmented venous signals (Fig. 8). The technique allows clearer depiction of the extent and distribution of collateral vessels, facilitating recognition of the characteristic cavernomatous pattern and improving differentiation from other periportal vascular or biliary structures. By providing real-time visualization of complex collateral venous pathways without the use of contrast agents, MV-Flow supports a more confident non-invasive ultrasound assessment of portal cavernoma and its hemodynamic features during routine abdominal examinations and follow-up.

#### Arteriovenous shunts and abnormal venous filling

**Fig. 9:**
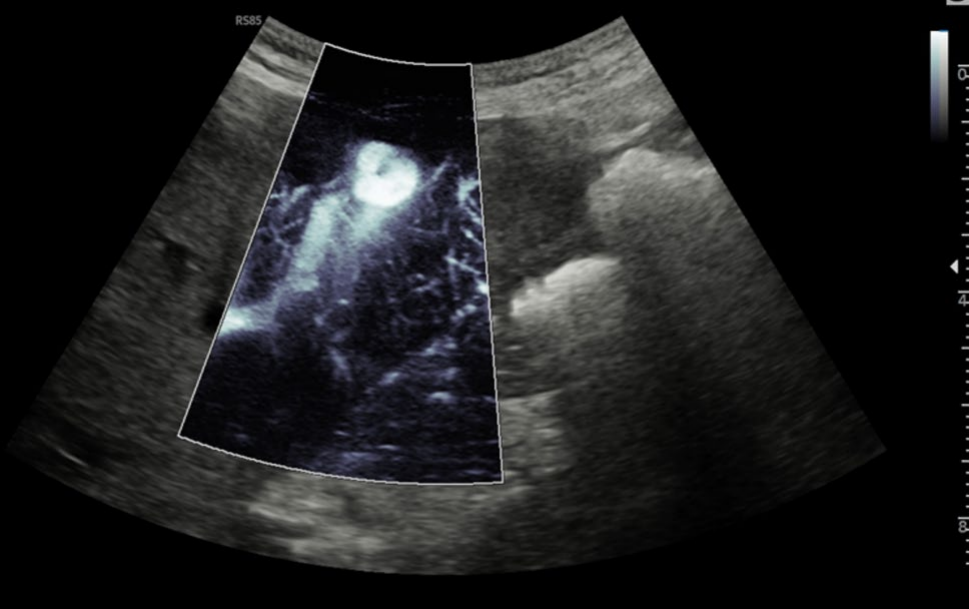
Intrahepatic arteriovenous fistula: MV-Flow imaging. MV-Flow imaging demonstrates an abnormal intrahepatic vascular communication characterized by a focal area of intense and heterogeneous flow signal with early venous filling, consistent with an intrahepatic arteriovenous fistula. The technique allows visualization of complex and multidirectional flow patterns along the abnormal vascular pathway, which are difficult to appreciate with conventional color Doppler ultrasound. Enhanced sensitivity to low-velocity and heterogeneous flow supports recognition of abnormal arteriovenous shunting and improves non-invasive ultrasound assessment of intrahepatic vascular malformations

Arteriovenous shunts represent an uncommon but clinically relevant cause of abnormal venous hemodynamics, characterized by direct communication between the arterial and venous systems. This condition results in early and heterogeneous venous filling, with complex and multidirectional flow patterns that may be challenging to interpret using conventional color and power Doppler ultrasound, particularly when flow velocities are low or spatially fragmented. In this setting, MV-Flow contributes to improved visualization of abnormal venous filling patterns by enhancing sensitivity to low-velocity and complex flow signals (Fig. 9). The technique allows clearer depiction of tortuous vascular pathways and venous structures involved in the shunt, facilitating recognition of abnormal arteriovenous connections and differentiation from physiological venous flow or motion-related artifacts. By providing real-time visualization of complex flow patterns without the use of contrast agents, MV-Flow supports the non-invasive ultrasound assessment of arteriovenous shunts, complementing conventional Doppler techniques and guiding further diagnostic evaluation with contrast-enhanced ultrasound or cross-sectional imaging.

### Arterial flow alterations and dynamic disorders

Arterial conditions associated with altered or dynamic flow patterns represent a challenging field for Doppler ultrasound assessment. Current international guidelines on peripheral arterial and aortic diseases emphasize the importance of non-invasive imaging techniques, including Doppler ultrasound, as first-line tools for diagnosis and follow-up [1, 2]. In these settings, blood flow may be slow, intermittent, multidirectional, or significantly influenced by respiratory movements, leading to suboptimal visualization with conventional color and power Doppler techniques. Motion artifacts, angle dependency, and limited sensitivity to low-velocity flow may further hinder accurate depiction of complex arterial hemodynamics. In this context, MV-Flow supports improved visualization of complex and dynamic arterial flow patterns, facilitating a more comprehensive qualitative assessment of arterial hemodynamics during routine abdominal ultrasound examinations.

#### Splenic artery aneurysm

**Fig. 10:**
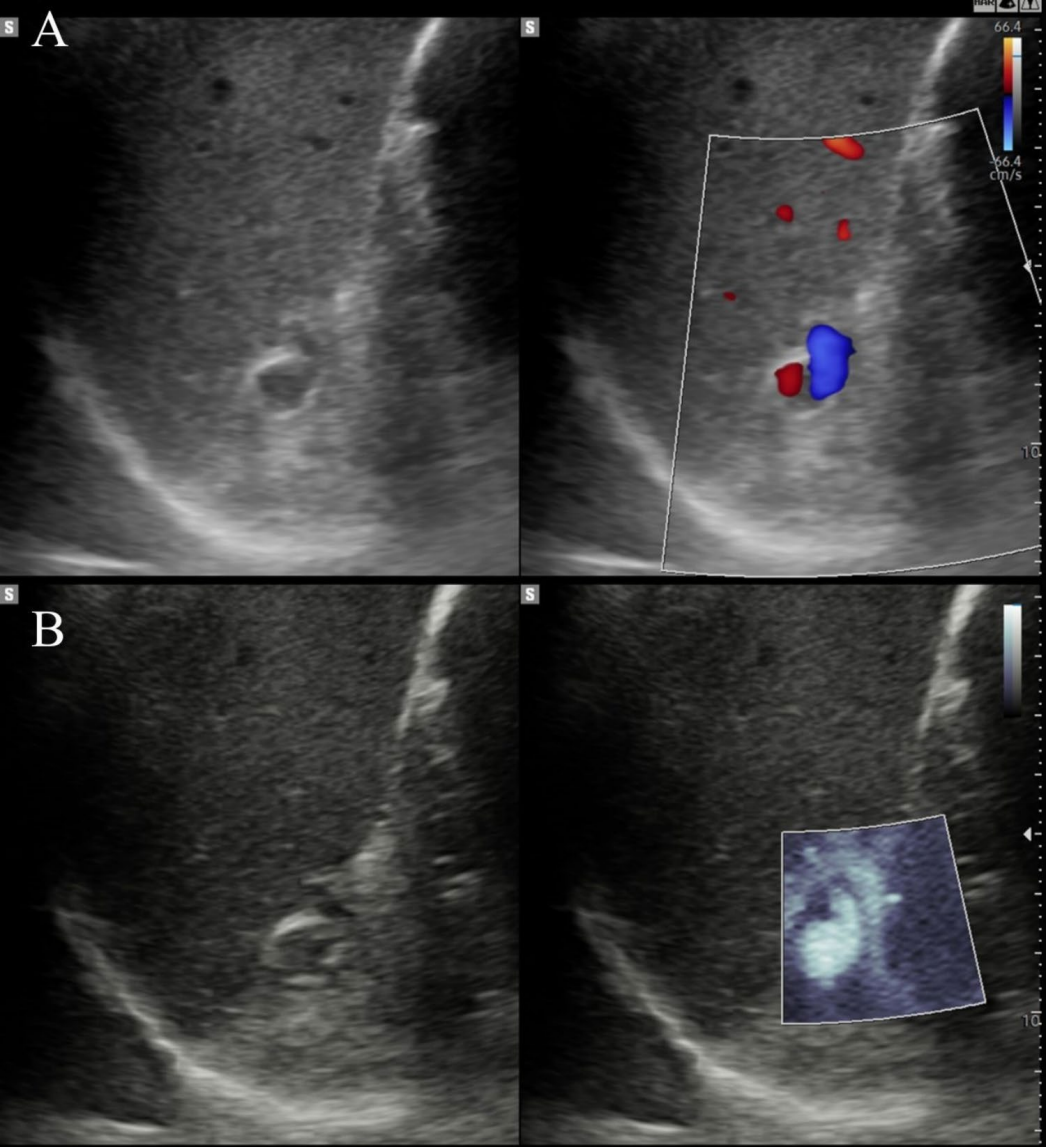
Small splenic artery aneurysm: comparison between conventional color Doppler and MV-Flow imaging. **A** B-mode ultrasound demonstrates a small, round, anechoic lesion adjacent to the splenic artery, without specific morphological features allowing confident characterization. Conventional color Doppler ultrasound shows limited and discontinuous intralesional flow signals, providing incomplete depiction of aneurysmal blood flow. **B** MV-Flow imaging clearly delineates low-velocity intraluminal blood flow within the aneurysmal sac, allowing more accurate visualization of residual perfused areas and aneurysm filling. The enhanced sensitivity of MV-Flow to slow and complex arterial flow improves sonographic assessment of small splenic artery aneurysms that may be underestimated with conventional Doppler techniques

Small splenic artery aneurysms may present with low-velocity or heterogeneous intraluminal blood flow, particularly in partially thrombosed or slow-flowing aneurysmal sacs. Conventional color Doppler ultrasound may incompletely depict intraluminal flow because of blooming artifacts or insufficient sensitivity to slow and complex flow patterns. In this setting, MV-Flow allows clearer delineation of intraluminal blood flow within the aneurysmal sac, improving visualization of residual perfused areas and supporting non-invasive ultrasound assessment of small splenic artery aneurysms (Fig. 10).

#### Endoleak after endovascular aortic repair

**Fig. 11:**
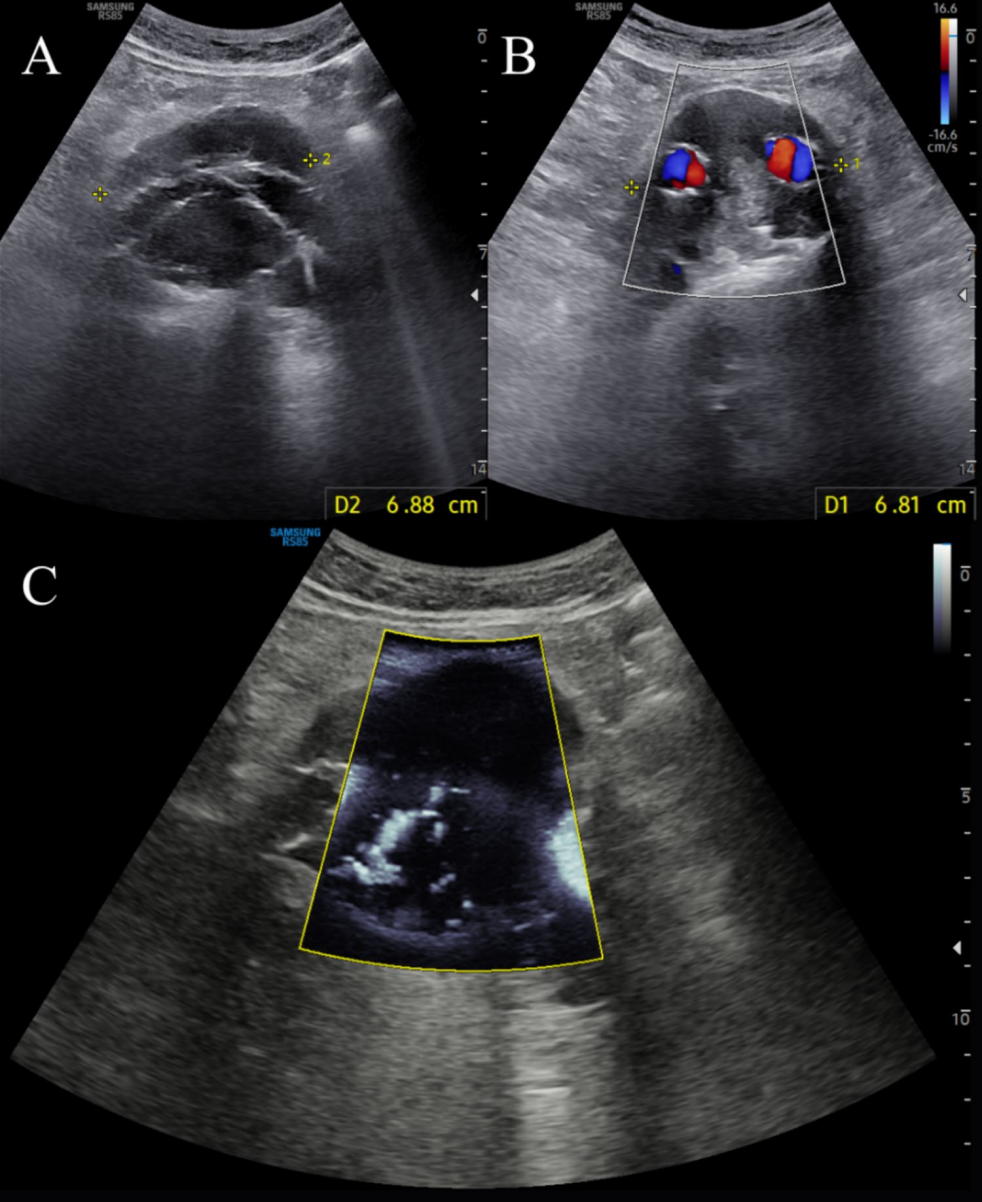
Type II endoleak after endovascular aortic repair. **A** B-mode ultrasound demonstrates a hypoechoic aneurysm sac surrounding the endograft. **B** Conventional color Doppler ultrasound demonstrates blood flow signals confined to the endograft lumen, without evidence of abnormal peri-prosthetic flow within the aneurysm sac.** C** MV-Flow imaging clearly depicts multiple low-velocity peri-prosthetic flow signals within the aneurysm sac, consistent with a type II endoleak, highlighting slow-flow vascular channels not readily appreciable with conventional Doppler techniques

Endoleaks are a common complication following endovascular aortic repair (EVAR) and are classified according to the source and mechanism of aneurysm sac reperfusion. Among them, type II endoleak is the most frequent and is characterized by low-velocity retrograde blood flow from collateral vessels, such as lumbar arteries or the inferior mesenteric artery, into the aneurysm sac. According to current ESC and ACC/AHA guidelines on aortic diseases, imaging plays a central role in post-procedural surveillance after EVAR, with particular emphasis on the detection of endoleaks and monitoring of aneurysm sac behavior [1, 2]. In this context, ultrasound represents a valuable non-invasive tool for follow-up, and advanced techniques such as MV-Flow may improve the detection of low-velocity endoleaks in selected patients. Owing to its slow-flow nature and susceptibility to motion artifacts related to respiration or bowel movements, type II endoleak may be challenging to detect using conventional color or Power Doppler ultrasound. MV-Flow enhances visualization of peri-prosthetic slow-flow signals within the aneurysm sac, facilitating identification of subtle vascular channels and residual arterial inflow that may not be readily appreciable with standard Doppler techniques (Fig. 11). Recent meta-analyses have reported high diagnostic performance of microvascular flow imaging techniques for the detection of type II endoleaks compared with CT angiography, thus showing a pooled sensitivity and specificity of 0.91 (CI: 0.82–0.96) and 0.98 (CI: 0.94–1.00), respectively [21]. Although results may vary across studies and platforms, these findings suggest that MV-Flow may represent a valuable non-invasive tool for EVAR surveillance, particularly in patients requiring repeated imaging. Visualization of characteristic low-velocity flow patterns within the aneurysm sac may therefore support a more confident and radiation-free follow-up strategy after endovascular repair.

#### Aortic dissection

**Fig. 12:**
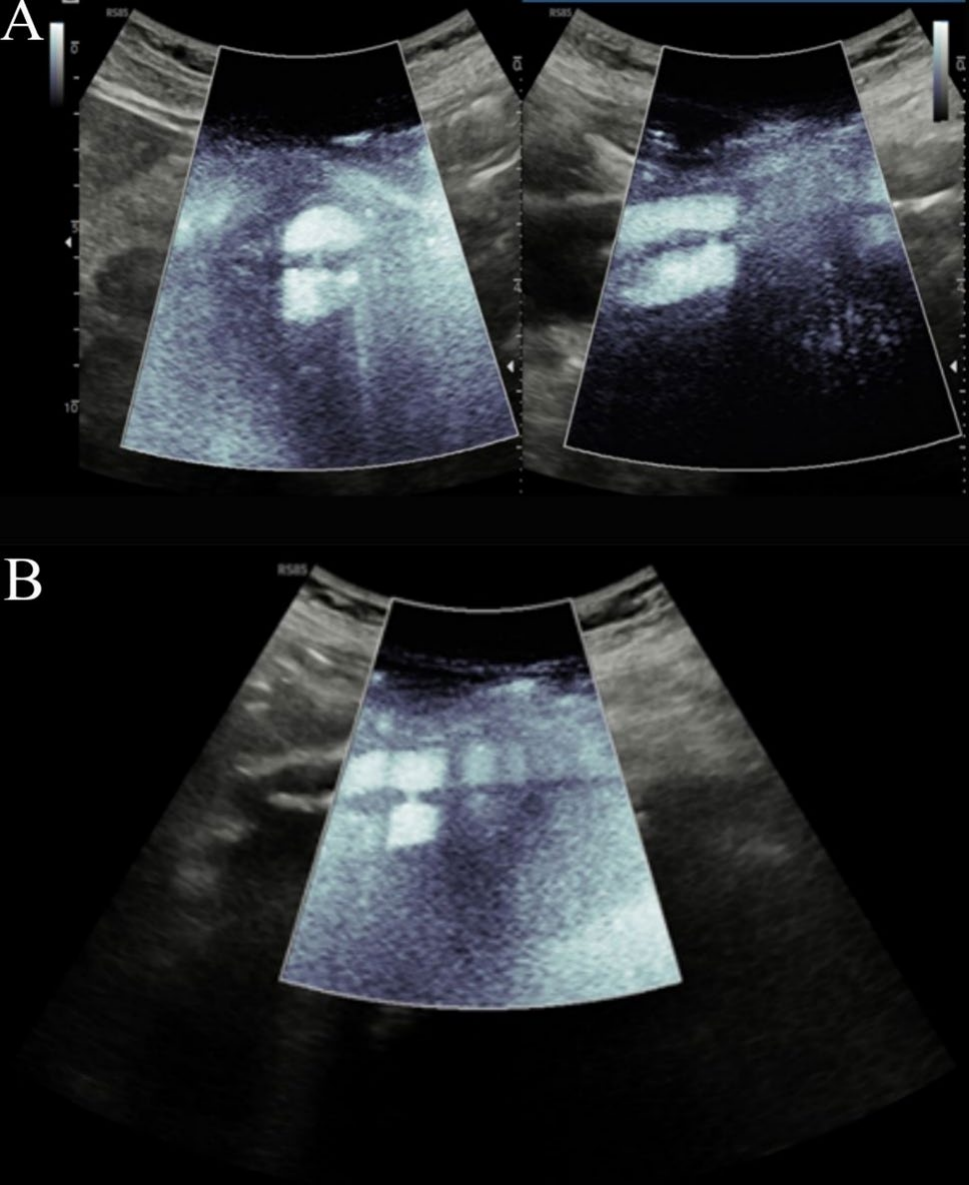
Aortic dissection: MV-Flow imaging of double-lumen perfusion. **A**, **B** MV-Flow imaging demonstrates simultaneous low-velocity blood flow within both the true and false lumens of the dissected aorta, confirming perfusion of the double-lumen configuration. MV-Flow allows also direct visualization of a focal communication tract between the true and false lumen, depicting a low-velocity flow pathway that is difficult to appreciate with conventional Doppler techniques. The enhanced sensitivity of MV-Flow to slow and complex intraluminal flow supports detailed qualitative assessment of aortic dissection hemodynamics during non-invasive ultrasound evaluation

In aortic dissection, arterial flow is characterized by complex hemodynamics related to the presence of true and false lumens, with variable flow direction, velocity, and communication between the two channels [22]. Conventional Doppler techniques may be limited in depicting subtle flow exchanges or small communication tracts between lumens, especially when flow velocities are low. MV-Flow enhances visualization of complex intraluminal flow patterns by depicting low-velocity flow within both lumens and allowing identification of communication pathways between the true and false lumen, supporting ultrasound evaluation of aortic dissection in selected clinical settings (Fig. 12).

#### Dunbar syndrome

**Fig. 13:**
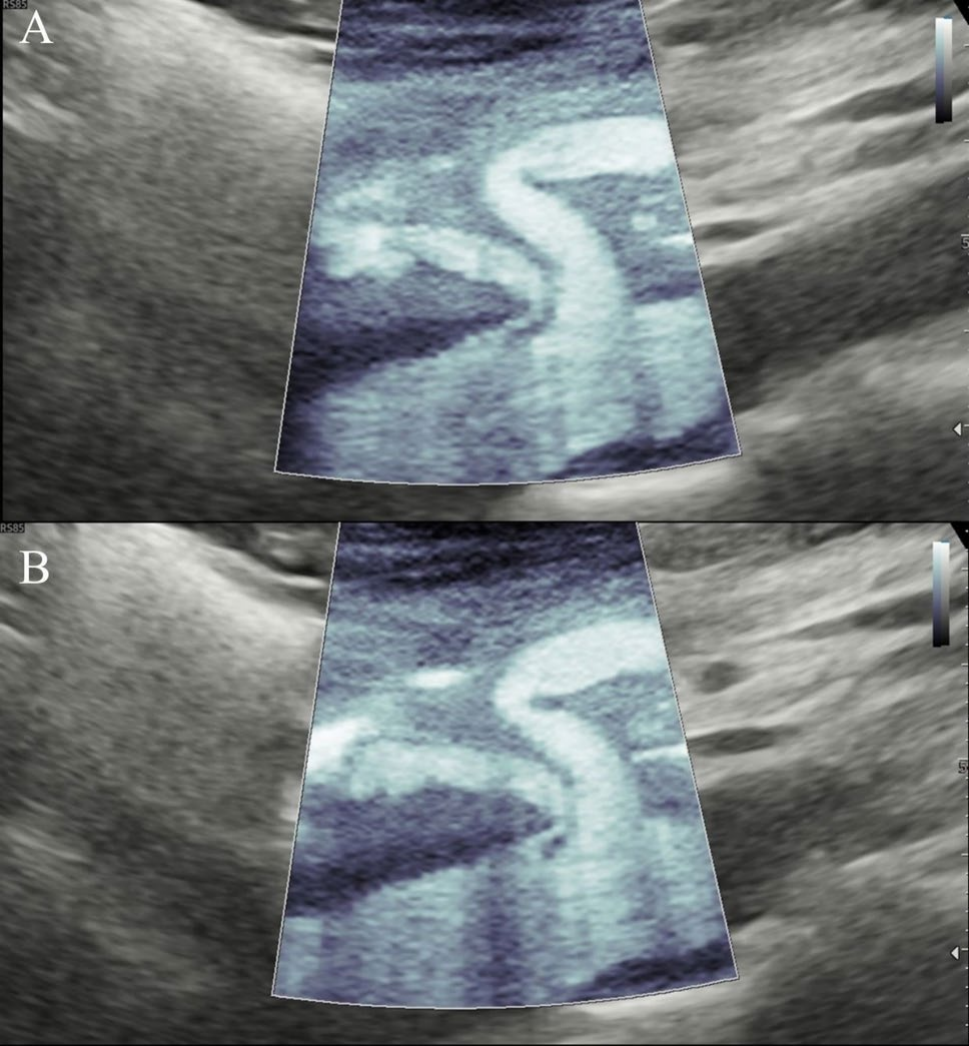
Dunbar syndrome: MV-Flow imaging during different respiratory phases. **A** MV-Flow imaging acquired during inspiration demonstrates a tortuous arterial flow pattern of the celiac trunk with partial relief of extrinsic compression. **B** MV-Flow imaging acquired during expiration shows a more pronounced arterial narrowing with marked flow distortion and redistribution, consistent with dynamic extrinsic compression by the median arcuate ligament. The ability of MV-Flow to depict low-velocity and complex flow patterns allows direct visualization of respiratory-dependent hemodynamic changes that may not be readily appreciable with conventional Doppler techniques

Dunbar syndrome is characterized by dynamic extrinsic compression of the celiac trunk, resulting in variable arterial flow alterations that are strongly influenced by respiratory movements. The hemodynamic behavior of this condition is often complex, with fluctuating degrees of flow reduction and post-stenotic turbulence that may challenge conventional Doppler assessment. MV-Flow contributes to the evaluation of Dunbar syndrome by improving the visualization of dynamic arterial flow patterns and subtle changes in vascular perfusion that may not be consistently appreciable with standard Doppler techniques. By directly depicting respiratory-dependent arterial flow alterations, MV-Flow supports the sonographic evaluation of suspected Dunbar syndrome within a comprehensive, non-invasive abdominal ultrasound examination (Fig. 13).

### Acute pyelonephritis

**Fig. 14:**
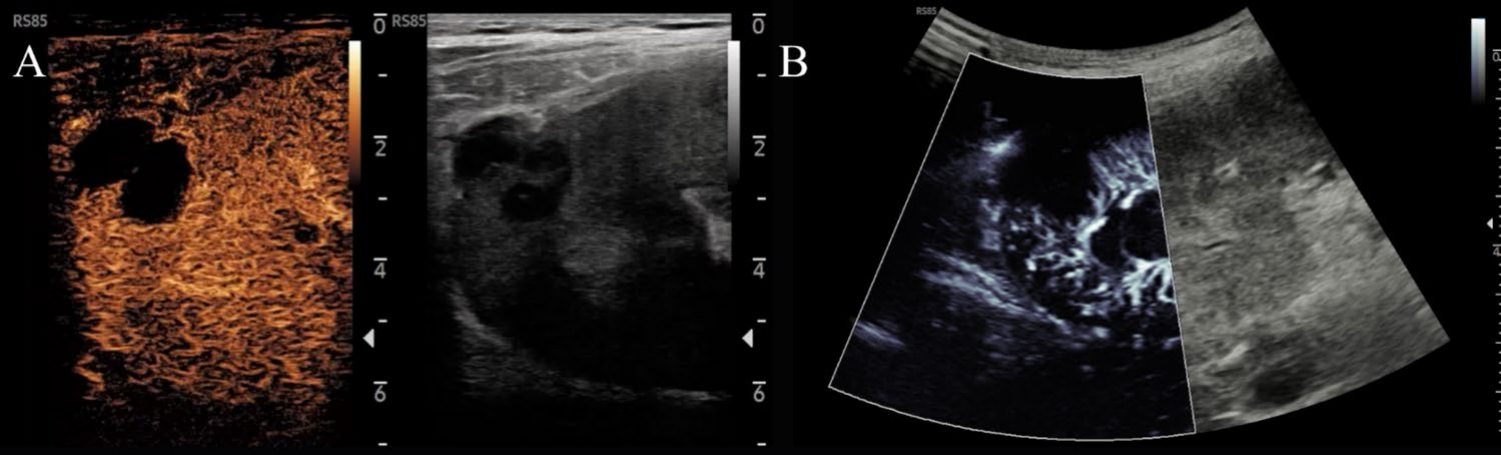
Acute pyelonephritis: concordance between CEUS and MV-Flow. **A** Contrast-enhanced ultrasound (CEUS) demonstrates a focal area of reduced parenchymal enhancement, consistent with inflammatory perfusion impairment in acute pyelonephritis. **B** MV-Flow imaging shows a corresponding area of reduced microvascular perfusion within the renal parenchyma, displaying a perfusion pattern concordant with CEUS findings, without the use of contrast agents

**Fig. 15:**
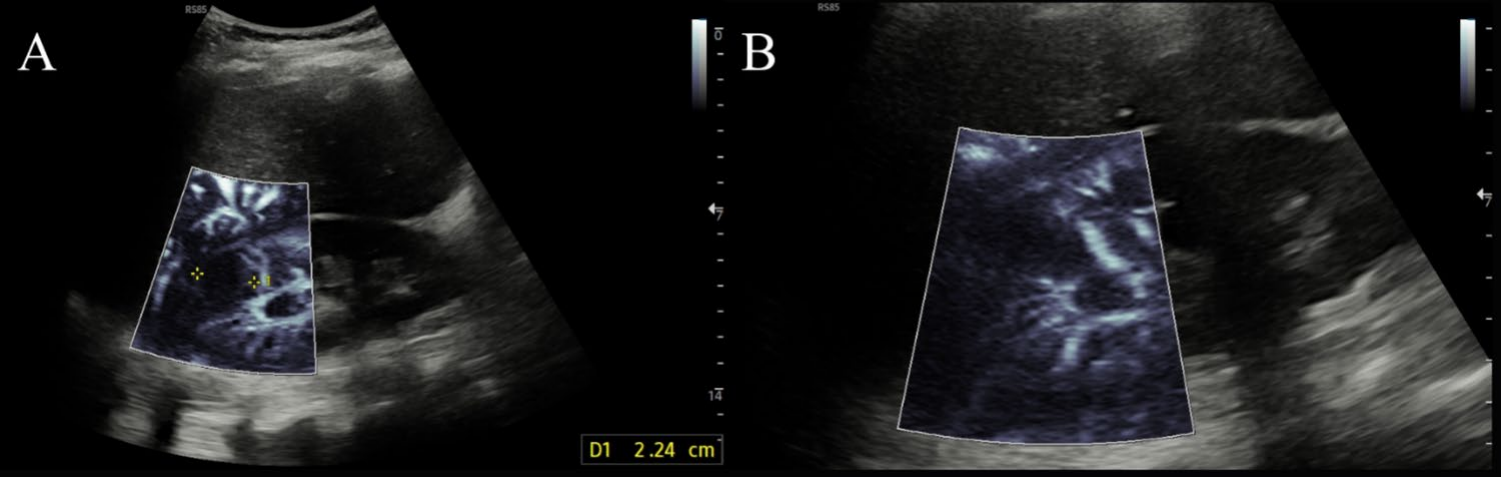
Acute pyelonephritis during pregnancy – MV-Flow imaging. **A–B** MV-Flow imaging demonstrates focal alterations of renal microvascular perfusion consistent with acute pyelonephritis in a pregnant patient. Areas of reduced and heterogeneous parenchymal perfusion are clearly visualized without the use of ultrasound contrast agents. In this clinical setting, MV-Flow allows assessment of inflammatory perfusion abnormalities when contrast-enhanced ultrasound is contraindicated, supporting a safe and non-invasive sonographic evaluation

**Fig. 16:**
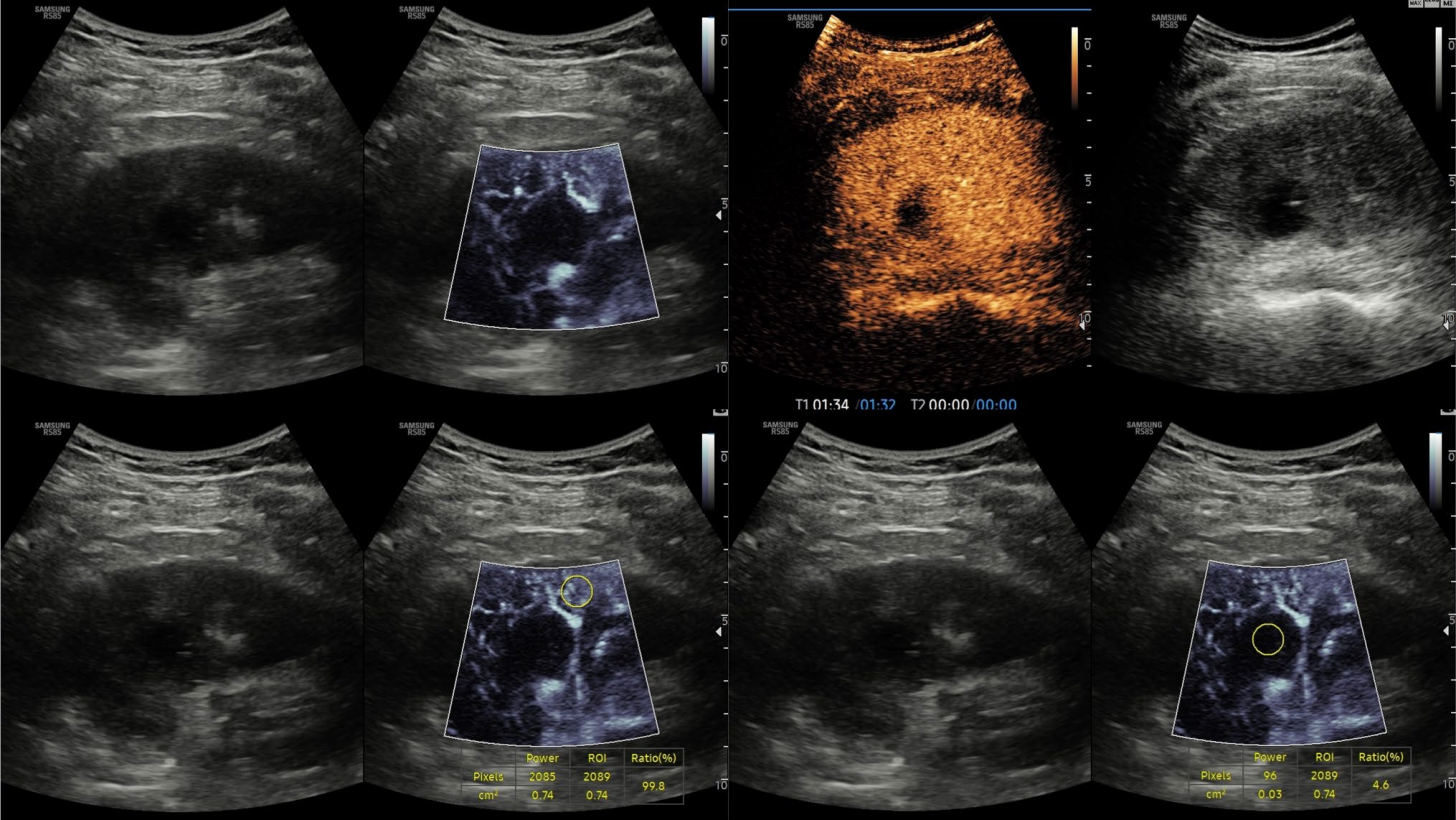
Renal abscess evaluated with multiparametric ultrasound. Conventional B-mode imaging demonstrates a hypoechoic lesion, while contrast-enhanced ultrasound (CEUS) shows a non-enhancing central cavity consistent with necrosis. Microvascular imaging (MV-Flow) highlights peripheral vascular signals surrounding the lesion. Quantitative vascularity index analysis confirms a marked reduction of perfusion within the abscess (4.6%) compared with adjacent normal renal parenchyma (99.8%)

Acute pyelonephritis is characterized by regional alterations in renal parenchymal perfusion related to inflammatory edema and microvascular involvement. Conventional Color and Power Doppler ultrasound are often inadequate for detecting these perfusion abnormalities, as inflammatory changes frequently occur in the absence of macroscopic vascular alterations or detectable flow disturbances. MV-Flow enables visualization of renal microvascular perfusion patterns by depicting areas of reduced or heterogeneous blood flow within the renal parenchyma. In this setting, MV-Flow findings show good concordance with contrast-enhanced ultrasound (CEUS) [23] in identifying perfusion defects associated with acute pyelonephritis, supporting its role as a reliable, non-invasive imaging tool. Importantly, in clinical scenarios in which the use of ultrasound contrast agents is contraindicated, such as pregnancy, MV-Flow allows assessment of parenchymal perfusion without contrast administration (Figs. 14 and 15). This capability supports the sonographic evaluation of acute pyelonephritis in selected patients, expanding diagnostic possibilities within a comprehensive and safe abdominal ultrasound examination. In addition to qualitative assessment, microvascular imaging techniques may provide quantitative parameters such as the Vascularity Index (VI), defined as the ratio between flow signal pixels and total pixels within a region of interest [11] (Fig. 16). This metric may allow a more objective evaluation of parenchymal perfusion in inflammatory conditions, although its clinical application remains under investigation. Recent evidence suggests that three-dimensional MV-Flow imaging may enable quantitative assessment of microvascular perfusion through parameters such as VI, FI, and VFI, which have demonstrated reproducibility and potential clinical value in the evaluation of both normal and abnormal organ perfusion, including fetal renal conditions [24].

## Pitfalls and limitations

Despite its advantages, MV-Flow presents some limitations that should be considered during clinical application. As a Doppler-based technique, MV-Flow remains inherently angle-dependent and may be affected by suboptimal insonation angles. In addition, the high sensitivity to slow flow makes the technique susceptible to motion-related artifacts, including flash artifacts caused by respiration, patient movement, or cardiac pulsatility, which may lead to potential misinterpretation. Therefore, artifacts may occasionally mimic true vascular signals, requiring careful interpretation in conjunction with B-mode and conventional Doppler findings.

Image quality and flow depiction remain dependent on adequate acoustic windows and operator experience. MV-Flow provides qualitative information on flow patterns rather than quantitative hemodynamic measurements; therefore, it should not be used as a standalone technique for velocity assessment or grading of stenosis. In addition, the increased sensitivity to slow flow may occasionally lead to misinterpretation of noise or non-vascular signals, requiring careful correlation with B-mode imaging and conventional Doppler findings. For these reasons, MV-Flow should be considered a complementary tool integrated into a multiparametric ultrasound assessment, rather than a replacement for established Doppler techniques or contrast-enhanced ultrasound.

## Conclusions

MV-Flow represents a valuable addition to abdominal ultrasound by improving the visualization of low-velocity blood flow and microvascular perfusion in a wide range of clinical scenarios. Through enhanced depiction of flow patterns and vascular architecture, MV-Flow supports pattern recognition and increases diagnostic confidence in conditions that are challenging to assess with conventional Doppler techniques. Further standardization across vendors and validation in large-scale studies remain necessary. Although large-scale randomized studies are still lacking, MV-Flow is emerging as a valuable adjunct within multiparametric ultrasound. Its non-invasive and contrast-free nature makes it particularly appealing in clinical scenarios where contrast agents or ionizing radiation should be avoided, such as in pediatric and obstetric populations. The integration of advanced ultrasound techniques such as MV-Flow within a guideline-based diagnostic pathway may further enhance the role of ultrasound as a first-line, non-invasive imaging modality in abdominal vascular diseases.

## References

[1] Isselbacher EM, Preventza O, Hamilton Black Iii J et al (2022) ACC/AHA guideline for the diagnosis and management of aortic disease: a report of the American heart association/American college of cardiology joint committee on clinical practice guidelines. J Am Coll Cardiol 2022(80):e223–e393. 10.1016/j.jacc.2022.08.004 10.1016/j.jacc.2022.08.004 PMC9860464 36334952

[2] Mazzolai L, Teixido-Tura G, Lanzi S et al (2024) 2024 ESC guidelines for the management of peripheral arterial and aortic diseases. Eur Heart J 45:3538–3700. 10.1093/eurheartj/ehae179 39210722 10.1093/eurheartj/ehae179

[3] Becciolini M, Brighenti A, Tiraferri V et al (2025) B-flow imaging in abdominal ultrasound: a pictorial essay. J Ultrasound 28:1017–1030. 10.1007/s40477-025-01068-x 40839300 10.1007/s40477-025-01068-x PMC12675890

[4] Aziz MU, Eisenbrey JR, Deganello A et al (2022) Microvascular flow imaging: a state-of-the-art review of clinical use and promise. Radiology 305:250–264. 10.1148/radiol.213303 36165794 10.1148/radiol.213303 PMC9619200

[5] Leroy H, Wang LZ, Jimenez A et al (2025) Assessment of microvascular flow in human atherosclerotic carotid plaques using ultrasound localization microscopy. EBioMedicine 111:105528. 10.1016/j.ebiom.2024.105528 39729884 10.1016/j.ebiom.2024.105528 PMC11733184

[6] Mao Y, Mu J, Zhao J et al (2022) The comparative study of color doppler flow imaging, superb microvascular imaging, contrast-enhanced ultrasound micro flow imaging in blood flow analysis of solid renal mass. Cancer Imaging 22:21. 10.1186/s40644-022-00458-2 35505388 10.1186/s40644-022-00458-2 PMC9066849

[7] Cantisani V, Radzina M, Dietrich CF et al (2025) EFSUMB guidelines on multiparametric ultrasound thyroid nodule evaluation: PART II. Ultraschall Med. 10.1055/a-2761-1329s39909049

[8] Boccatonda A, Schiavone C, Serra C et al (2025) Gallbladder polyps: ultrasound diagnosis, updated guidelines, and clinical management. Ultraschall Med. 10.1055/a-2655-8601 40962117 10.1055/a-2655-8601

[9] Lu R, Meng Y, Zhang Y et al (2017) Superb microvascular imaging (SMI) compared with conventional ultrasound for evaluating thyroid nodules. BMC Med Imaging 17:65. 10.1186/s12880-017-0241-5 29281991 10.1186/s12880-017-0241-5 PMC5745911

[10] Cannella R, Pilato G, Mazzola M et al (2023) New microvascular ultrasound techniques: abdominal applications. Radiol Med 128:1023–1034. 10.1007/s11547-023-01679-6 37495910 10.1007/s11547-023-01679-6 PMC10473992

[11] Kikuchi S, Kayama K, Kawada Y et al (2024) Evaluation of renal circulation in heart failure using superb microvascular imaging, a microvascular flow imaging system. J Med Ultrason 51(2):283–292. 10.1007/s10396-023-01397-6 10.1007/s10396-023-01397-6 PMC13013226 38236503

[12] Wachsberg RH (2003) B-flow, a non-Doppler technology for flow mapping: early experience in the abdomen. Ultrasound Q 19:114–122. 10.1097/00013644-200309000-00002 14571159 10.1097/00013644-200309000-00002

[13] Járay Á, Farkas PI, Botz B (2025) The, “snow globe” sign: a microvascular flow imaging artifact in cystic lesions, and the role of dispersed internal debris. Abdom Radiol (NY). 10.1007/s00261-025-05328-6 41417077 10.1007/s00261-025-05328-6 PMC13269463

[14] Ruan L, Liu H, Xiang H et al (2024) Application of O-RADS US combined with MV-Flow to diagnose ovarian-adnexal tumors. Ultrasonography 43:15–24. 10.14366/usg.23061 38061878 10.14366/usg.23061 PMC10766884

[15] Bae JS, Lee JM, Jeon SK et al (2020) Comparison of MicroFlow Imaging with color and power Doppler imaging for detecting and characterizing blood flow signals in hepatocellular carcinoma. Ultrasonography 39:85–93. 10.14366/usg.19033 31759383 10.14366/usg.19033 PMC6920623

[16] Han H, Ji Z, Huang B et al (2023) The preliminary application of simultaneous display of contrast-enhanced ultrasound and micro-flow imaging technology in the diagnosis of hepatic tumors. J Ultrasound Med 42:729–737. 10.1002/jum.16111 36217761 10.1002/jum.16111

[17] Wang B, Yang J, Tang YL et al (2024) The value of microvascular Doppler ultrasound technique, qualitative or quantitative shear-wave elastography of breast lesions for predicting axillary nodal burden in patients with breast cancer. Quant Imaging Med Surg 14:408–420. 10.21037/qims-23-445 38223085 10.21037/qims-23-445 PMC10784034

[18] Giammalva GR, Viola A, Maugeri R et al (2022) Intraoperative evaluation of brain-tumor microvascularization through MicroV IOUS: a protocol for image acquisition and analysis of radiomic features. Cancers (Basel). 10.3390/cancers14215335 36358754 10.3390/cancers14215335 PMC9656308

[19] Wang Y, Feng H, Wei L et al (2026) Ultra-microangiography for evaluating Crohn’s disease activity in pediatric patients: a prospective study. Ultraschall Med. 10.1055/a-2771-2690 41386290 10.1055/a-2771-2690

[20] Boccatonda A, Campello E, Tallarico V et al (2025) Splanchnic venous thrombosis in patients with acute cholecystitis: a case series and review of literature. J Ultrasound 28:551–561. 10.1007/s40477-025-01029-4 40447979 10.1007/s40477-025-01029-4 PMC12496303

[21] Czeczelewski M, Kopyto E, Kuczyńska M et al (2024) Diagnostic accuracy of microvascular flow imaging ultrasound for endoleak detection after endovascular aortic aneurysm repair: a systematic review and meta-analysis. Pol J Radiol 89:e414–e419. 10.5114/pjr/190502 39257925 10.5114/pjr/190502 PMC11384215

[22] Boccatonda A, Brighenti A, Tiraferri V et al (2025) POCUS for acute abdominal pain: practical scan protocols on gastrointestinal diseases and an evidence review. J Ultrasound 28:851–871. 10.1007/s40477-025-01088-7 41023565 10.1007/s40477-025-01088-7 PMC12675880

[23] Boccatonda A, Brighenti A, Tiraferri V et al (2025) Bedside CEUS: a feasible option to consider. Eur J Intern Med 136:146–147. 10.1016/j.ejim.2025.02.001 39919919 10.1016/j.ejim.2025.02.001

[24] Huang C, Zhang L, Jiang Y et al (2024) Evaluation of normal and abnormal fetal renal microvascular flow characteristics of three-dimensional MV-flow imaging. Early Hum Dev 199:106149. 10.1016/j.earlhumdev.2024.106149 39547115 10.1016/j.earlhumdev.2024.106149

